# Bibliometric analysis of peer-reviewed literature on climate change and human health with an emphasis on infectious diseases

**DOI:** 10.1186/s12992-020-00576-1

**Published:** 2020-05-08

**Authors:** Waleed M. Sweileh

**Affiliations:** Department of Physiology, Pharmacology/Toxicology, Division of Biomedical Sciences, College of Medicine and Health Sciences, An-Najah National University, Nablus, Palestine

**Keywords:** Climate change, Health, Infectious diseases, Bibliometric analysis

## Abstract

**Background:**

Assessing research activity is important for planning future protective and adaptive policies. The objective of the current study was to assess research activity on climate change and health with an emphasis on infectious diseases.

**Method:**

A bibliometric method was applied using SciVerse Scopus. Documents on climate change and human health were called “health-related literature” while documents on climate change and infectious diseases were called “infection-related literature”. The study period was from 1980 to 2019.

**Results:**

The search query found 4247 documents in the health-related literature and 1207 in the infection-related literature. The growth of publications showed a steep increase after 2007. There were four research themes in the health-related literature: (1) climate change and infectious diseases; (2) climate change, public health and food security; (3) heat waves, mortality, and non-communicable diseases; and (4) climate change, air pollution, allergy, and respiratory health. The most frequently encountered pathogens/infectious diseases in the infection-related literature were malaria and dengue. Documents in infection-related literature had a higher *h*-index than documents in the health-related literature. The top-cited documents in the health-related literature focused on food security, public health, and infectious diseases while those in infection-related literature focused on water-, vector-, and mosquito-borne diseases. The European region had the highest contribution in health-related literature (*n* = 1626; 38.3%) and infection-related literature (*n* = 497; 41.2%). The USA led with 1235 (29.1%) documents in health-related literature and 365 (30.2%) documents in infection-related literature. The *Australian National University* ranked first in the health-related literature while the *London School of Hygiene & Tropical Medicine* ranked first in the infection-related literature. International research collaboration was inadequate. Documents published in the *Environmental Health Perspectives* journal received the highest citations per document. A total of 1416 (33.3%) documents in the health-related literature were funded while 419 (34.7%) documents in the infection-related literature were funded.

**Conclusion:**

Research on climate change and human health is on the rise with research on infection-related issues making a good share. International research collaboration should be funded and supported. Future research needs to focus on the impact of climate change on psychosocial, mental, innovations, policies, and preparedness of health systems.

## Background

Climate change refers to long-term statistical shifts of the earth’s climate system that result in new climate patterns [1]. Over the past century, industrial activities have led to long-term changes in the climate system that included global warming, flooding, and drought [2]. The *Paris Agreement*, an agreement within the United Nations Framework Convention on Climate Change (UNFCCC) signed in 2016, represents an opportunity for all countries to implement measures to reduce, combat, and adapt to climate change [3–6]. Combating and reducing climate change is an important goal of the sustainable development goals (SDGs) which states “Take urgent action to combat climate change and its impacts” [7]. The implications of climate change on human health have led the World Health Organization (WHO) to declare climate change as one of the top ten global health threats in 2019 [8]. Climate change is negatively affecting human lives by changing the quality of air, water, and food supply [9–11]. It is estimated that between 2030 and 2050, climate change will cause approximately 250,000 additional deaths per year and 2–4 billion USD loss per year by 2030 [12]. These devastating economic and health consequences require national and international planning to slow down climate change and to build resilient health systems that can tackle these changes [13]. The effects of climate change are global and diverse [11, 14, 15]. However, the impact on developing countries with limited resources and weak health systems will be more obvious [16–20].

Climate change has affected the epidemiology and pattern of both communicable and non-communicable diseases [21]. For example, changes in temperature have serious adverse effects on the pattern and incidence of infectious diseases [22]. Global warming favors the survival and transmission of causative pathogens or vectors of the causative agent [23–27]. Climate change influences the dynamics of vector-borne, water-borne, food-borne, rodent-borne, and air-borne infectious diseases [28, 29]. Furthermore, a recent study predicted that climate change might worsen antimicrobial resistance [30]. The study indicated that a spike in temperature of 10 C was linked with a 4.2% increase in antibiotic resistance to *E.coli*, which can trigger serious food poisoning; a 2.7% increase in *Staphylococcus aureus*, which can cause skin infections and food poisoning; and a 2.2% increase in *Klebsiella pneumoniae*, which can cause pneumonia. The spread of antimicrobial resistance is believed to have a serious negative global impact on human health [30]. A study predicted that if antimicrobial resistance is not addressed, then by 2050, 10 million people will die because of antimicrobial resistance [31]. Many recent studies predicted that serious and emerging infectious diseases could appear or get worsened by climate change [8, 32]. It is expected that the epidemiology and geography of many infectious diseases will change due to climate variability [32]. For example, climate change will be an important factor for the spread of Lassa virus in Western Africa [33]. Droughts are expected to increase the epidemics of West Nile Virus globally [34]. Higher incidence of cases of Chikungunya and Zika virus infections in Brazil have been attributed to areas with more frequent rainfall and severe droughts [35]. Climate change and increased global temperatures have been associated with an increase in the probability of Rift Valley Fever, cholera and malaria [36]. The expected rapid spread of infectious diseases with climate change in the presence of antimicrobial resistance might cause global mass fatalities [37–39]. The WHO considers climate change as a new threat to global health. This threat is compounded by globalization and modernization which can allow novel diseases to travel rapidly as what happened in the case of COVID-19 [40]. Actually, the emergence of entirely novel diseases, like COVID-19, reintroduced the discussion of the impact of climate change on infectious diseases carried by wild animals or mosquitos and transmitted to humans.

Assessing research activity on climate change helps identify the national and international contribution to this field, the hot themes discussed by researchers, and research gaps in the field. Climate change is a broad scientific topic and assessing research activity on climate change, in general, might not be very helpful. Therefore, in the current study, the research activity of climate change on human health with an emphasis on infectious diseases was investigated. Emphasis on infections was made due to suspected serious global outbreaks of infectious diseases such as dengue, Ebola, and others [41–45]. Second, investigating research activity on climate change will help understand the type of infections mostly affected by climate change. Third, research on climate change helps in developing appropriate protective measures and preparedness plans for certain infectious diseases in certain geographical areas. Fourth, research on climate change comes as a response to calls made by international organizations such as the WHO on the importance of the impact of climate change on health and infectious diseases. Based on the argument mentioned above and based on calls for papers made by certain specialized and prestigious journals in the field of public health and infectious diseases, the current study was undertaken to analyze the research aspects and research activity of climate change and human health with an emphasis on infectious diseases. The method used to display the research pattern and research activity on a certain topic is the bibliometric analysis which has been commonly used recently in various health topics [46–53]. Climate change has diverse effects that include aquatic organisms, forests, animals, and humans. The use of a bibliometric analysis is a suitable methodology to identify the volume and growth pattern of literature focusing on humans for further analysis related to health and infectious diseases. Furthermore, bibliometric analysis is a suitable methodology to spot important research themes and active researchers and research institutions for future funding and planning.

A literature search using well-known databases and search engines such as Scopus database and Google Scholar revealed that there were at least ten bibliometric studies on climate change and its effects on various aspects on ecology or agriculture or adaptation [54–61]. However, no bibliometric research papers were published on climate change and health or climate change and infectious diseases. Therefore, the current study will establish the first baseline data on this topic for future comparisons and for policymakers to draw plans on climate change and human health with an emphasis on infectious diseases.

## Methodology

### Database

The first step in any bibliometric study is to decide on the appropriate database to be used to retrieve the relevant documents. In the current study, SciVerse Scopus was used to accomplish the objective of the study. Scopus is larger than Web of Science and has more than 23,000 indexed journals in all scientific fields [62]. Scopus is 100% inclusive of Medline and therefore, it is far better than Medline. Furthermore, the export of data from Scopus to other programs is easy to perform. Scopus offers two methods of search; a basic and an advanced search in which complex and long search queries can be made to accomplish the objective with high validity. Scopus allows for search using terms in titles or titles/abstracts or name of the journal or name of the author or affiliation.

### Search strategy

The second challenge in any bibliometric study is to build a valid search query that will retrieve as many documents as possible but with minimum irrelevant (false-positive) results. In the field of climate change, many keywords could be used. However, in the current study, the authors reviewed many articles published as “systematic reviews” or “bibliometric analyses” to build a search query for climate change [22, 63–67]. The keywords used included, but not restricted to, the followings “climat* chang*” or “greenhouse effect” or “changing climate” or “global warming” or “extreme weather” or “climate variability” or “greenhouse gas” or “rising temperature” or “heat waves”. Other non-specific keywords were also used but under certain constraints. For example, keywords such as flood, drought, temperature, warm*, rain*, and “air pollution” were used under the condition that a phrase related to “climat* chang*” was also present in the title/abstract of the same document. The keyword “air pollution/air pollutant*” was used with restrictions because air pollution and climate change are closely related but are not the same. Therefore, documents on air pollution within the context of climate change were included [68]. Actually, in systematic review studies on climate change, the keyword air pollution was not included [69, 70]. Similarly, in previously published blibliometric studies on climate change, air pollution was not included in the search terms [70]. In Scopus, the quotation marks were used to retrieve the exact words while the asterisk was used as a wild card.

In the current study, the authors developed an extensive and comprehensive search query to retrieve all potential documents focusing on climate change and human health. The keywords used to retrieve health-related documents included, but not restricted to, health, respirat*, mood, cardiac, heart, hunger, “food *security”, pregnancy, asthma, infect*, “infectious”, “vector-borne disease”, “water-borne disease”, and many others. The search query was built mainly on title search to make sure that the retrieved documents are obviously and directly related to human health. Additional file 1 included all keywords and steps used to retrieve documents on climate change and health. Documents retrieved from the search query on human health and climate change were called **“health-related literature”**.

For documents related to infectious diseases, the authors used the same search query stated above but with all possible keywords related to infection/infectious diseases, pathogens, and vectors transmitting pathogens to humans. Documents retrieved for infectious diseases were called **“infection-related document”.** Details on the search query are shown in Additional File 1.

### Validation

Validation of the search queries was based on two approaches. In the first approach, the top 50 cited documents in the health- and infection-related literature were reviewed to make sure that they fit within the scope of climate change and health or climate change and infectious diseases. This approach was adopted to eliminate false-positive results by excluding documents focusing on the impact of climate change on certain plants or animals or any document irrelevant to human health. The second approach was based on comparing the actual number of articles for each author, obtained from his/her personal Scopus profile, with the number of articles obtained by the search query for active authors. The comparison was made using the *Pearson correlation test*. A significant and strong correlation is indicative of a high validity of the search query and the absence of missing results. This approach was previously used in several bibliometric studies [46].

### Bibliometric indicators

Data in the retrieved literature was exported to Microsoft Excel. The exported data included annual growth of publications, types of documents, languages, countries, authors, institutions, journals, citations, and funding agencies. The retrieved literature was also exported to VOSviewer program [71] to create network visualization maps. The strength of international research collaboration was presented as Total Link Strength (TLS) which is automatically given by VOSviewer upon mapping research activity of selected countries. The TLS is proportional to the extent of international research collaboration where higher TLS value indicates greater collaboration. Bibliometric indicators were presented as top ten active ones. For annual growth, Statistical Package for Social Sciences (SPSS Statistics for Windows, Version 24.0. Armonk, NY: IBM Corp.) was used to draw the annual growth of publications. For geographical distribution of documents, the WHO regional classification was used: the region of the Americas (AMRO), the European region (EURO), the Western Pacific Region (WPRO), the Eastern Mediterranean region (EMRO), the South-Eastern Asia region (SEARO), and African region (AFRO).

The quality of publications was measured by the number of citations and h-index [72] while the quality or impact of the journal was measured using the quartile ranking of journals obtained from Scimago journal rank [73]. Journals in the Q1 rank are considered to have the highest impact. The study period was from 1980 to 2019. All citation analysis and data export were carried out on the same day (April 14, 2020) to avoid misinterpretation.

## Results

### Volume, types, and growth of publications

**Fig. 1:**
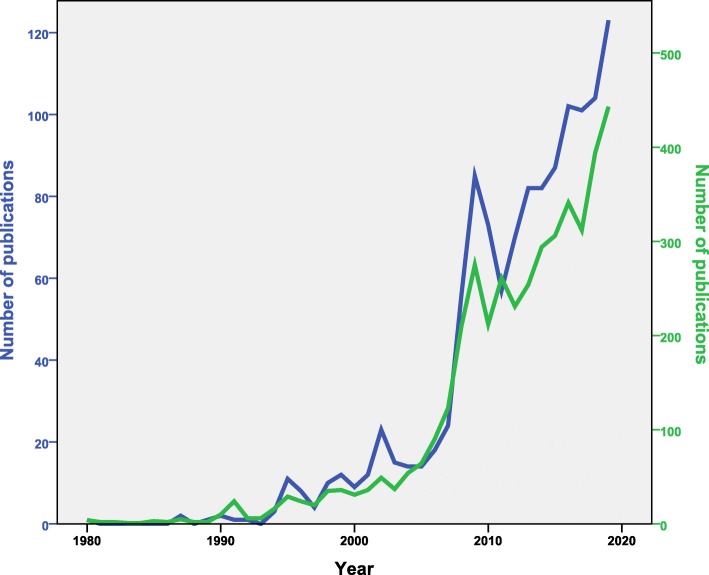
Annual growth of documents in the health- and infection-related literature (1980–2019). Green line represents the annual growth of documents in the health-related literature while the blue line represents the annual growth of documents in the infection-related literature. The graph was created by SPSS program using dual y-axis for comparative purposes.

The search query found 4247 documents on health-related literature and 1207 documents on infection-related literature. Therefore, infection-related literature constituted 28.4% of the health-related literature. Retrieved documents were of different types (Table 1**)**. Research articles constituted 62.2% (*n* = 2675) of the health-related literature and 68% (*n* = 821) of the infection-related literature. There was a larger percentage of editorials (*n* = 303; 7.1%) in the health-related literature compared with that in the infection-related literature (*n* = 37; 3.1%). The annual growth of publications in the health-related literature was low in the 1980s and 1990s but showed a steep increase after 2007. The annual growth pattern of documents in the infection-related literature followed the same pattern. However, the annual growth of documents in infection-related literature was relatively faster than that of the health-related literature. Figure 1 shows the growth of publications in the health- and infection-related literature depicted in dual-axis for easy comparison.

**Table 1:** Types of documents on health- and infection-related literature (1980–2019)

**Health-related documents**			**Infection-related documents**		
**Type of document**	**Frequency**	**%** ***N*** **= 4247**	**Type of document**	**Frequency**	**%** ***N*** **= 1207**
Article	2657	62.6	Article	821	68.0
Review	677	15.9	Review	225	18.6
Editorial	303	7.1	Note	44	3.6
Note	263	6.2	Editorial	37	3.1
Letter	204	4.8	Letter	32	2.7
Conference Paper	68	1.6	Conference Paper	26	2.2
Short Survey	67	1.6	Short Survey	21	1.7
Undefined^a^	8	0.2	Undefined	1	0.1

Infection-related documents: documents on climate change and infectious diseases

Health-related documents: documents on climate change and health

^a^Undefined documents represent documents that are not yet categorized by Scopus

### Research themes in health-related literature

**Fig. 2:**
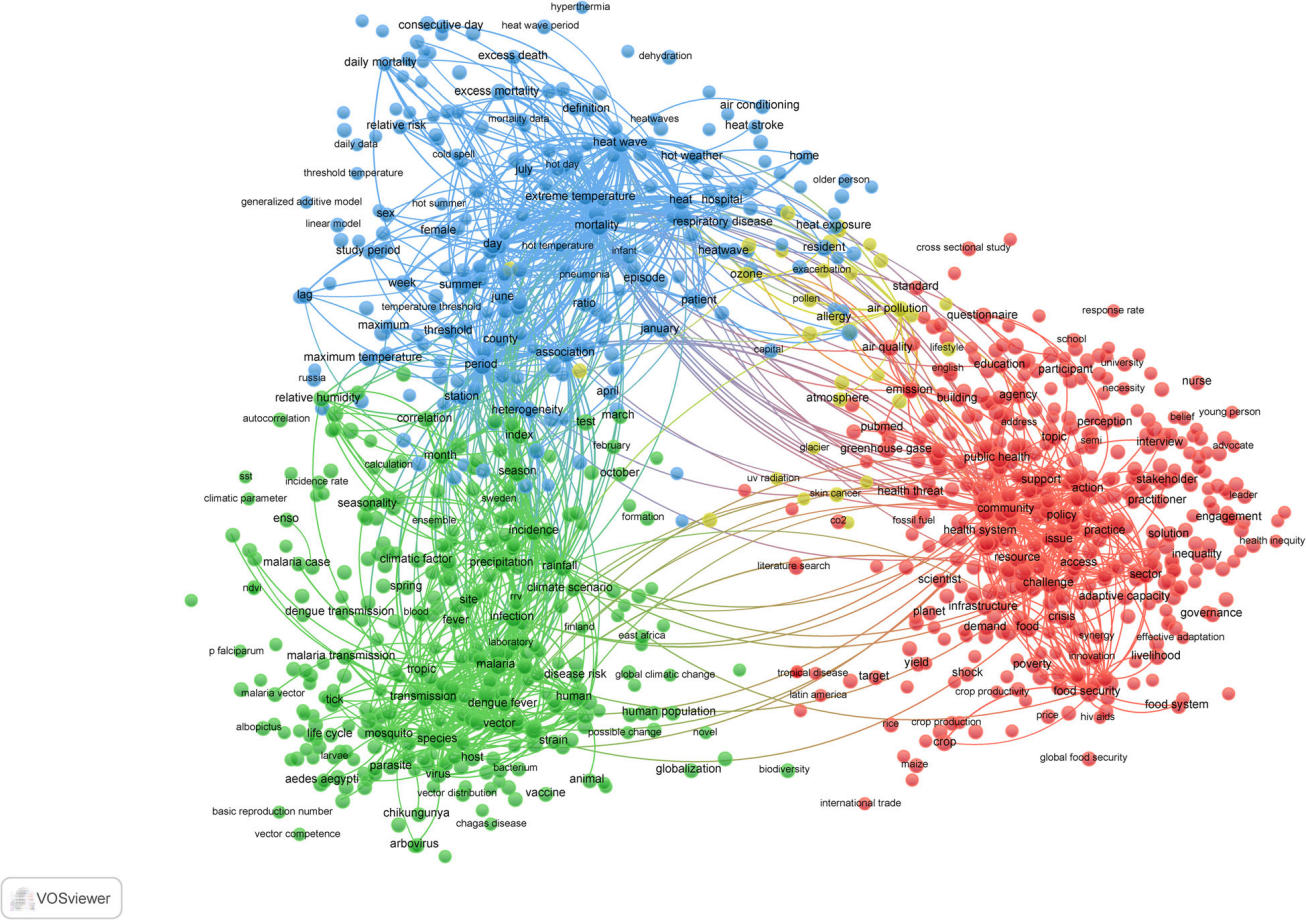
Network visualization map of terms in title/abstract fields of documents in the health-related literature. The minimum occurrences of included terms was10. The map shows four clusters representing four research themes. Nodes with similar color represent a cluster of related terms. The map was created by VOSviewer

Mapping the most frequent terms in title/abstract fields of documents in the health-related literature with a minimum occurrence of 10 gave 992 terms distributed in four clusters representing four main research themes (Fig. 2):

1. The first cluster (red) included 420 items and focused on the following topics arranged alphabetically: adaptation, climate change, food production, food security, public health, health policy, healthcare system, and psychology.
2. The second cluster (green) included 315 terms and focused on the following topics arranged alphabetically: *Aedes aegypti*, *Aedes albopictus*, anopheles, arbovirus, arthropod, Chagas disease, chikungunya, climate change, dengue, hantavirus, influenza, ixodes ricinus, Japanese encephalitis, leptospirosis, leishmaniasis, Lyme disease, Lyme borreliosis, malaria, mosquito, public health, rift valley fever, ross river virus, schistosomiasis, temperature change, ticks, vectors, West Nile virus, yellow fever, zoonosis, and zika.
3. The third cluster (blue) included 225 items and focused on the following topics arranged alphabetically: air temperature, cardiovascular disease, chronic obstructive pulmonary disease, climate change, dehydration, diabetes, elderly, extreme heat, heat waves, heat strokes, hyperthermia, hypertension, mortality, precipitation public health, pneumonia, respiratory diseases, salmonellosis, and stroke.
4. The fourth cluster (yellowish-green) included 32 terms and focused on the following topics: air pollution, air pollutants, atmosphere, allergy, allergic respiratory diseases, asthma, cancer, ozone depletion, pollen allergy, and respiratory health.

### Pathogens and diseases encountered in infection-related literature

**Fig. 3:**
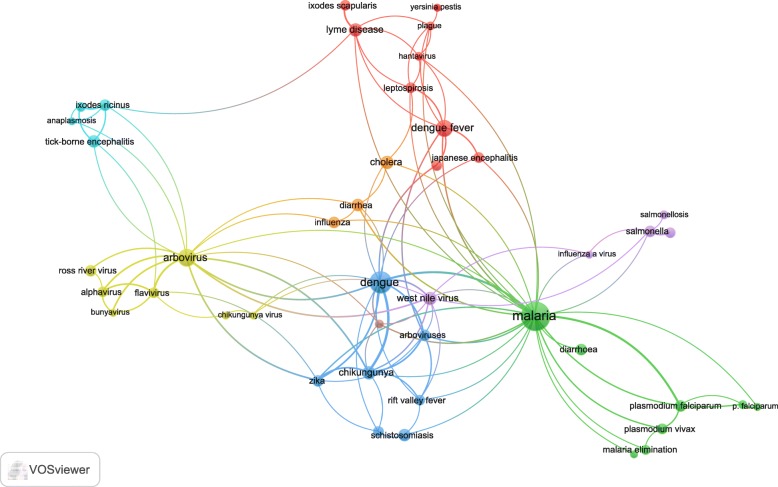
Network visualization map of author keywords in documents in the infection-related literature. Node size represents frequency of occurrence of the keyword. Larger node size represents keywords that are most frequent in the retrieved literature. The map was created by VOSviewer

Analysis of author keywords in infection-related literature indicated that malaria (112 occurrences), dengue (76 occurrences), and arboviruses (arthropod-borne viruses) (33 occurrences) were the most frequent infectious diseases/pathogens encountered (Table 2 and Fig. 3). Infection-related literature included 32 documents that discussed climate change and emerging infectious diseases such as malaria, dengue, Chikungunya, Lyme disease, West Nile virus, zika virus, arboviruses, flavivirus, hantavirus, tick-borne encephalitis, bluetongue virus, Cryptosporidiosis, rift valley fever, alpha virus and others.

**Table 2:** List of Infectious diseases/pathogens with minimum occurrences of five times in infection-related literature (1980–2019)

**Disease / Pathogen**^**a**^	**Number of occurrences in author keywords**
*Malaria*	112
*Dengue*	76
*Arbovirus*	33
*Diarrhea*	23
*Lyme disease*	18
*Chikungunya*	14
*Cholera*	12
*Salmonella*	12
*West Nile virus*	11
*Schistosomiasis*	11
*Plasmodium falciparum*	11
*Influenza*	11
*Plasmodium vivax*	10
*Tick-borne Encephalitis*	9
*Ross River virus*	8
*Zika*	8
*Rift valley fever*	7
*Leptospirosis*	6
*Leishmaniasis*	6
*Japanese encephalitis*	6
*Campylobacter*	5
*Cercariae*	5
*Chagas disease*	5
*Hantaviruses*	5

^a^This list is not 100% inclusive of all pathogens or infections present in the retrieved literature. Please see result section and Fig. 3 for more details

### Distribution based on WHO regions

Analysis of the retrieved documents based on geographical region indicated that EURO had the highest contribution to both health-related literature (*n* = 1626; 38.3%) and infection-related literature (*n* = 497; 41.2%). The EMRO region had the least contribution to both the health- and infection-related literature (Table 3). There was a significant and strong correlation between the percentage contribution of each region to health- and infection-related literature (*p* < 0.001, r = 0.99).

**Table 3:** Contribution of each WHO regions to health- and infection-related literature (1980–2019)

**Region based on World Health Organization**	**Health-related literature**		**Infection-related literature**	
	**Frequency**	**%** ***N* = 4247**^**a**^	**Frequency**	**%** ***N* = 1207**^**a**^
Region of the Americas	1548	36.4	476	39.4
European Region	1626	38.3	497	41.2
Western Pacific Region	913	21.5	281	23.3
South-East Asia Region	232	5.5	75	6.2
African Region	253	6.0	119	9.9
Eastern Mediterranean Region	92	2.2	31	2.6

Infection-related documents: documents on climate change and infectious diseases

Health-related documents: documents on climate change and health

^a^the total percentage exceeded 100% due to overlap among different regions created by international research collaborations

### Active countries and international research collaboration

**Fig. 4:**
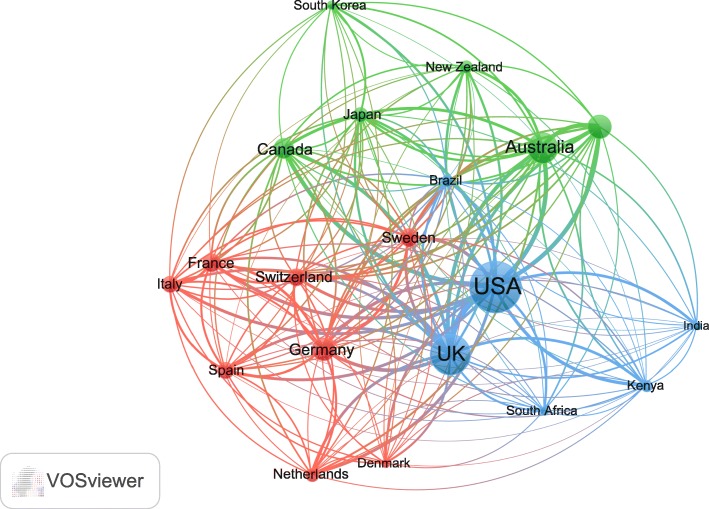
Network visualization map of international research collaboration among top 20 active countries in the health-related literature. The thickness of the connecting line (link strength) is proportional to the extent of research collaboration between the connected countries. Node size of each country represents the percentage of documents with international researchers. Larger node size is indicative of greater international research collaboration for that country. Similar color indicates close research interest. The map was created by VOSviewer

Table 4 shows the top ten active countries in publishing documents in health- and infection-related literature. The list of active countries in publishing documents in health- and infection-related literature was dominated by European countries. However, the USA led with 1235 (29.1%) documents in health-related literature and 365 (30.2%) documents in infection-related literature. The USA also had the highest percentage of documents with international researchers followed by the UK and Australia. Mapping research collaboration in the health-related literature for top 20 active countries yielded three main clusters with the USA and the UK located in the middle of the map (Fig. 4). The strongest collaboration was between the USA and the UK (link strength = 102) followed by that between the USA and China (link strength = 63). The red cluster in the map included seven European countries with similar research interests in the field of climate change and health. The USA and the UK shared similar research interests with most countries on the map since both were located in the middle of the map.

**Table 4:** Top ten active countries in publishing health- and infection-related literature (1980–2019)

**Health-related literature**						**Infection-related literature**					
**Rank**	**Country**	**Frequency**	**%** ***N* = 4247**	**C/D**	**TLS**	**Rank**	**Country**	**Frequency**	**%** ***N* = 1207**	**C/D**	**TLS**
1st	United States	1235	29.1	40.1	579	1st	United States	365	30.2	47.2	169
2nd	United Kingdom	592	13.9	39.8	435	2nd	United Kingdom	160	13.3	49.1	127
3rd	Australia	512	12.1	30.8	294	3rd	Australia	146	12.1	34.2	91
4th	Germany	264	6.2	26.6	230	4th	Germany	82	6.8	22.1	46
5th	Canada	248	5.8	28.5	194	5th	China	81	6.7	19.8	68
6th	China	234	5.5	24.8	234	6th	France	63	5.2	22.5	40
7th	France	198	4.7	36.5	177	7th	Canada	57	4.7	45.9	36
8th	Italy	151	3.6	46.5	145	8th	Netherlands	51	4.2	60.0	46
9th	India	137	3.2	12.1	59	9th	Italy	48	4.0	36.4	46
10th	Spain	124	2.9	39.3	142	9th	Sweden	48	4.0	44.7	35

C/D = number of citations per document

TLS = total link strength, a measure of the extent of international research collaboration

Infection-related literature: documents on climate change and infectious diseases

Health-related literature: documents on climate change and health

### Quality of publications

For the health-related literature, documents published by researchers from Italy ranked first in the number of citations per document (46.5) followed by those published by researchers from the USA (40.1) and the UK (39.3). For infection-related literature, documents published by researchers from the Netherlands ranked first in the number of citations per document (60.0) followed by documents published by researchers from the UK (49.1) and the USA (47.2).

### Citation analysis

Documents in the health-related literature received 95,684 citations, 22.5 citations per document, a median of 6, a range of 0–1481, and *h*-index of 133. The infection-related literature received 36,631 citations, a mean of 30.3 citations per document, a median of 12, a range of 0–588 and *h*-index of 92. The top ten cited documents in health-related literature focused on the impact of climate change on food security, public health, and infections [74–83]. In the infection-related literature, the top ten cited documents discussed water-, vector-, and mosquito-borne diseases as well as general effects of climate change on infectious diseases, particularly malaria and dengue [75–77, 84–90].

### Active institutions/organizations

The list of top active institutions/organizations for the health-related literature was dominated by Australian and American institutions with *Australian National University* ranking first with 129 (3.0%) documents (Table 5). For the infection-related literature, the list of the top ten active institutions/organizations was dominated by Australian academic institutions. However, *London School of Hygiene & Tropical Medicine* ranked first with 32 (2.7%) documents. The list included two non-academic institutions/organization; the WHO and the Chinese Centers for Disease Control and Prevention.

**Table 5:** Top ten active institutions/organizations in publishing health- and infection-related literature (1980–2019)

**Health-related literature (*N* = 4247)**				
**Rank**^**a**^	**Institutions/Organizations**	**Frequency**	**%**	**Country**
1st	Australian National University	129	10.7	Australia
2nd	London School of Hygiene & Tropical Medicine	128	10.6	UK
3rd	World Health Organization	79	6.5	WHO
4th	Centers for Disease Control and Prevention	67	5.6	USA
5th	Queensland University of Technology QUT	60	5.0	Australia
5th	University of Oxford	60	5.0	UK
7th	University of Washington, Seattle	59	4.9	USA
8th	Umea University	57	4.7	Sweden
9th	The University of Adelaide	56	4.6	Australia
10th	Columbia University in the City of New York	53	4.4	USA
10th	University of Queensland	53	4.4	Australia
**Infection-related literature (*N* = 1207)**				
1st	London School of Hygiene & Tropical Medicine	32	2.7	UK
2nd	Queensland University of Technology QUT	31	2.6	Australia
3rd	University of Oxford	30	2.5	UK
4th	Australian National University	29	2.4	Australia
5th	University of Queensland	24	2.0	Australia
5th	Chinese Center for Disease Control and Prevention	24	2.0	China
7th	The University of Adelaide	21	1.7	Australia
7th	World Health Organization	21	1.7	WHO
9th	Johns Hopkins Bloomberg School of Public Health	19	1.6	USA
9th	Monash University	19	1.6	Australia

^a^In ranking, two equally active institutions were given similar ranks and one position in the rank was skipped

Infection-related literature: documents on climate change and infectious diseases; Health-related literature: documents on climate change and health

### Active journals

For the health-related literature, *International Journal of Environmental Research and Public Health* was the leading journal with 184 (4.3%) documents. In the list of active journals, two journals were in the field of infection while the remaining were in the field of public health, environment, and general medicine (Table 6).

**Table 6:** Top ten active journals in publishing the health- and infection-related literature (1980–2019)

**Health-related literature (*N* = 4247)**						
**Rank**^**a**^	***Journal***	**Frequency**	**%**	**C/D**	**Country**	**Journal Rank**
1st	*International Journal of Environmental Research and Public Health*	184	4.3	11.4	Switzerland	Q2
2nd	*Environmental Health Perspectives*	102	2.4	78.3	United States	Q1
3rd	*Lancet*	78	1.8	42.6	United Kingdom	Q1
4th	*BMJ Clinical Research Ed*	66	1.6	2.2	United Kingdom	–
5th	*Climatic Change*	57	1.3	31.4	Netherlands	Q1
6th	*Parasites and Vectors*	56	1.3	24.9	United Kingdom	Q1
7th	*Plos One*	51	1.2	25.2	United States	Q1
8th	*Plos Neglected Tropical Diseases*	46	1.1	34.7	United States	Q1
9th	*American Journal of Public Health*	44	1.0	50.3	United States	Q1
10th	*Global Health Action*	40	0.9	25.5	United Kingdom	Q1
**Infection-related literature (1207)**						
1st	Parasites And Vectors	56	4.6	23.8	United Kingdom	Q1
2nd	Plos Neglected Tropical Diseases	47	3.9	32.3	United States	Q1
3rd	Malaria Journal	40	3.3	27	United Kingdom	Q1
4th	Epidemiology and Infection	33	2.7	23.3	United Kingdom	Q2
5th	Plos One	32	2.7	30.2	United States	Q1
6th	International Journal of Environmental Research and Public Health	21	1.7	15.4	Switzerland	Q2
7th	American Journal of Tropical Medicine and Hygiene	20	1.7	50.6	United States	Q1
7th	Environmental Health Perspectives	20	1.7	100.5	United States	Q1
9th	Emerging Infectious Diseases	16	1.3	53.9	United States	Q1
9th	Science of the Total Environment	16	1.3	28.1	Netherlands	Q1

^a^In ranking, two equally active journals were given similar ranks and one position in the rank was skipped. C/D = number of citations per document. Q = Quartile

Infection-related literature: documents on climate change and infectious diseases. Health-related literature: documents on climate change and health

For the infection-related literature, *Parasites and Vectors* journal was the leading journals with 56 (4.6%) documents. However, documents published in *Environmental Health Perspectives* received the highest (100.5) citations per document. Of the top active journals, five were in the field of infection, four in the field of environmental and public health, and one miscellaneous. In general, documents in infection-related literature received a higher number of citations per document than documents in health-related literature. For example, documents about infections in the *Environmental Health Perspective* received 100.5 citations per document while documents about climate change and health in the same journal received 78.3 citations per document.

### Active authors

Table 7 shows the top ten active authors of health- and infection-related literature. Researchers from Europe, North America, Australia, and China dominated both lists. The top active author for the health-related literature was from the USA while the top active author for the infection-related literature was from China. In total, 5552 author names participated in publishing the infection-related literature, an average of 4.6 authors per document. One hundred and thirty-nine (11.5%) documents were single-authored.

**Table 7:** Top ten active authors in publishing the health- and infection-related literature (1980–2019)

**Health-related Literature (*N* = 4247)**				
**Rank**^**a**^	**Author**	**Frequency**	**%**	**Country affiliation**
1st	Ebi, K.L.	73	1.7	United States
2nd	McMichael, A.J.	49	1.2	United Kingdom
3rd	Tong, S.	41	1.0	China
4th	Haines, A.	39	0.9	United Kingdom
5th	Bi, P.	37	0.9	Australia
6th	Patz, J.A.	29	0.7	United States
7th	Campbell-Lendrum, D.	25	0.6	World Health Organization
7th	Semenza, J.C.	25	0.6	Sweden
9th	Kinney, P.L.	23	0.5	United States
9th	Rocklöv, J.	23	0.5	Sweden
**Infection-related Literature (*N* = 1207)**				
1st	Tong, S.	21	1.7	China
1st	Bi, P.	21	1.7	Australia
3rd	Liu, Q.	19	1.6	China
4th	Hu, W.	14	1.2	Australia
4th	Semenza, J.C.	14	1.2	Sweden
6th	Patz, J.A.	13	1.1	United States
7th	Hay, S.I.	11	0.9	United States
7th	Reiter, P.	11	0.9	France
9th	Ebi, K.L.	10	0.8	United States
9th	Hashizume, M.	10	0.8	Japan
9th	Ogden, N.H.	10	0.8	Canada
9th	Pascual, M.	10	0.8	United States
9th	Randolph, S.E.	10	0.8	United Kingdom
9th	Suk, J.E.	10	0.8	European CDC (Sweden)
9th	Weinstein, P.	10	0.8	Australia

^a^In ranking, two equally active authors were given similar ranks and one position in the rank was skipped

Infection-related literature: documents on climate change and infectious diseases. Health-related literature: documents on climate change and health

### Funding

For health-related literature, a total of 1416 (33.3%) documents were funded. The National Natural Science Foundation in China was the most active funding agency (*n* = 62; 1.5%) followed by the National Institutes of Health (NIH; USA) and National Science Foundation (NSF; USA) (Table 8). For the infection-related literature, analysis showed that 419 (34.7%) documents were funded. The NIH (USA) was the most active in funding (*n* = 43; 3.6%) followed by NSF (USA) (*n* = 29; 2.4%). The WHO was listed as one of the top ten active funding agencies infection-related literature.

**Table 8:** Top ten active funding agencies in publishing the health- and infection-related literature (1980–2019)

**Health-related literature (*N* = 4247)**				
**Rank**^**a**^	**Funding agency**	**Frequency**	**%**	**Country affiliation**
1st	National Natural Science Foundation of China	62	1.5	China
2nd	National Institutes of Health	58	1.4	United States
2nd	National Science Foundation	58	1.4	United States
4th	U.S. Environmental Protection Agency	29	0.7	United States
5th	National Basic Research Program of China (973 Program)	27	0.6	China
6th	Centers for Disease Control and Prevention	26	0.6	United States
6th	European Commission	26	0.6	Europe
8th	Natural Environment Research Council	23	0.5	United States
9th	National Institute of Environmental Health Sciences	21	0.5	United States
9th	Wellcome Trust	21	0.5	UK
**Infection-related literature (*N* = 1207)**				
1st	National Institutes of Health	43	3.6	United States
2nd	National Science Foundation	29	2.4	United States
3rd	National Institute of Allergy and Infectious Diseases	15	1.2	United States
4th	Wellcome Trust	12	1.0	UK
5th	National Natural Science Foundation of China	11	0.9	WHO
6th	Centers for Disease Control and Prevention	10	0.8	Canada
7th	National Basic Research Program of China (973 Program)	10	0.8	China
7th	International Development Research Centre	9	0.7	Canada
9th	World Health Organization	9	0.7	USA
9th	European Commission	8	0.7	USA

^a^In ranking, two equally active funding agencies were given similar ranks and one position in the rank was skipped

Infection-related literature: documents on climate change and infectious diseases. Health-related literature: documents on climate change and health

## Discussion

The current study was carried out to give a snap shot of research on climate change on human health with an emphasis on infectious diseases. The current study showed an increasing number of publications on climate change and health in the past decade. The gradual increase in the number of publications was parallel to international warning signals since the early 1980s about the impact of climate change on human health. The first major international conference on the greenhouse effect at Villach, Austria, warned that greenhouse gases will cause a rise of global mean temperature which is greater than any in man’s history [91]. In 1988, the United Nations (UN) created the Intergovernmental Panel on Climate Change (IPCC) to analyze and report on scientific findings. In 1992, Climate Change Convention, agrees to reduce emissions from industrialized countries to stop global warming [92]. In 1997, the Kyoto Protocol calls for cutting emissions from industrialized nations [93]. However, due to political and economic reasons in industrialized nations, the Koyoto protocol did not come into force until 2005. The IPCC fourth report issued in 2007 called for all countries to take adaptive measures to face climate changes [94].

The steep rise in the number of publications on climate change and human health coincided with the release of the fourth IPCC report which blamed humankind activities for the irreversible climate changes. In the face of increasing evidence of the impact of climate change on human health, the WHO took an active role in developing policies to minimize the impact of climate change on health. In 2015, the WHO Executive Board endorsed a new work plan on climate change and health that included raising awareness, endorse science and research on climate change, and support public health adaptive measures for climate change [95]. The timeline history, debate, negotiations, and conventions at the international levels affected both the volume and pattern of research on climate change and its impact on human health.

### Research themes

The current study indicated that there were four research themes on climate change and human health. These research themes were closely related. Of particular interest in the current study was the research theme focusing on climate change and infectious disease. However, there was a small cluster representing the interplay of climate change and air pollution in the context of human health. Air pollution is a complex subject and has fundamental effects on human health. In the current study, we focused on air pollution within the context of climate change and the resultant effect on human health. That is why the air pollution research theme was the smallest research theme as shown in the map. Both climate change and air pollution are global environmental problems that are closely related and considered as twins but they are not the same thing. Climate change is the global variation of the Earth’s climate which is accelerated by greenhouse gases caused by human activity. Carbon dioxide is the main gas contributing to climate change, but it is not harmful to human health. Air pollution is defined as the presence, in the air, of substances or particles that imply danger, damage or disturbance for humans, flora or fauna. The main sources of atmospheric contamination are gases that result mainly from emissions caused by the burning of fossil fuels emissions generated by transport, industrial processes, burning of forests, aerosol use, and radiation. Both climate change and air pollution are worsened by the burning of fuel, increasing the CO2 emissions which cause global warming. Meanwhile, the generation of other pollutants, such as nitrogen oxides (NO and NO2), sulfur oxides (SO2 and SO3) and particulate matter, is the main reason the air is contaminated [96, 97]. Climate variations affect air quality; air pollution can worsen climate change and both can directly or indirectly affect health [68]. The major and obvious health effect of climate change and air pollution is on respiratory health where both can exacerbate allergies and bronchial asthma [98]. The complex interactions between climate change and air quality is a new area of research that requires further investigation [99, 100].

The current study indicated that infections constituted a major theme of research on climate change and human health. Climate change and temperature rise affect the transmission and spread of many pathogens [101]. The current study showed that documents about malaria and dengue were among the top ten cited documents. Malaria was the most frequently encountered infectious disease affected by climate change. Malaria is a vector-borne disease that is sensitive to long-term climate change. For example, malaria epidemic risk increases around five-fold in the year after an El Niño event in India [102]. Researchers have developed mathematical models to forecast future climatic influences on infectious diseases. The model aims to apply the statistical equations to future climate scenarios in order to predict the actual distribution of the disease. These models have been applied to malaria and dengue fever [27, 76, 103–108]. The case incidence of dengue fever has multiplied 30-fold since the 1960s [109]. According to the WHO estimation, 30.0–54.7% of the world’s population (2.05–3.74 billion) is living in areas where dengue viruses can be transmitted [110].

### Key players in health- and infection-related research

The current study showed that more than two-thirds of the global publications came from the AMRO and EURO regions. There are many reasons for the leading role of these two regions. The presence of the US CDC, Euro CDC, and many other governmental and non-governmental research and academic institutions the field of public health and infectious diseases helped these two regions to make this tremendous and significant contribution. Second, the main funding agencies are located in these two regions. Third, infections have no borders and pathogens could travel with human migration waves which made Europe and North American regions in a critical geographic position to any infectious disease outbreak. Fourth, both regions have a great responsibility toward climate change since many of these changes were made by industrial activities. The current study showed that China was among the top ten active countries. The contribution of China might be underestimated because it is possible that most publications from China were published in national Chinese journals that are not indexed in Scopus. The same argument could be applied to other regions and countries with a limited number of peer-reviewed journals indexed in Scopus such as Russia or certain countries in South America. The current study showed that AFRO region made a greater contribution than either EMRO or SEARO region. A possible reason for the relatively higher contribution of the AFRO region is the strong research collaboration between certain African countries and the USA and the UK. Climate change in the AFRO region increased the number of people in Africa who are at risk of malaria [111, 112]. The increase in the number of mosquitoes increased the opportunity for both *Plasmodium falciparum* and *Plasmodium vivax* parasites to proliferate and place more people at risk of contracting malaria [113, 114]. Aside from malaria, the AFRO region is expected to suffer from hunger and food insecurity due to climate change [115, 116]. The climate change in the AFRO region is worsened by the weak economies, lack of resilient health systems, and lack of political stability in certain African regions.

The current study showed that the EMRO region had the least contribution despite that the region is expected to suffer from serious climate variations [117]. A systematic review on climate change and health in the EMRO region identified many knowledge and research gaps with research scarcity in this field [118]. The authors of the systematic review concluded that the impact of climate change on health is not recognized as a priority area by health researchers, health professionals and policymakers in the EMRO region. International research collaboration and funding are important for countries in the EMRO, AFRO, SEARO regions where effects of climate change are expected to be beyond their economic and research capabilities.

### Citation analysis

The current study showed that retrieved publications received a high number of citations suggestive of a large number of researchers who are interested in the topic. This could be attributed to the following reasons: First, the topic of climate change has caught the attention of scientists all over the world since the early 1980s with many national and international warning reports about the climate change. Second, the climate change is a multidisciplinary subject and therefore, researchers and scientists in public health, infectious diseases, nutrition, environmental health, ecology, and others were highly keen to investigate the subject and to have an input in this evolving topic. Third, the fact that the top ten active journals in publishing documents were influential in their field gave credibility and attracted a larger number of citations. Fourth, the leading role of the WHO as an international health agency played a positive role in raising the number of citations. Finally, the number of authors played a positive role in increasing the number of citations [119]. The current study indicated that infection-related documents received a higher number of citations and a higher h-index than documents in the health-related literature. This finding suggests that of the diverse health effects of climate change, its impact on the epidemiology and emerging infections receives the greatest scientific attention. This is due to the high and immediate risks of emerging and re-merging infections on global health. The h-index of the infection-related literature was higher than that reported for strongyloidiasis literature [120], epidermal parasitic skin diseases [121], antimalarial drug resistance [122], but lower than that on campylobacter or carbapenem resistance [123, 124].

### Research gaps and future directions

The current study emphasizes the importance of certain future research directions in the field of climate change and human health. First, it is of great importance to introduce the concept of climate change and its relation to human health in medical education. Future physicians and other healthcare workers need to understand the dynamics of human health, particularly infectious diseases, in relation to climate change. Climate change made the spread and the emergence of new infectious disease a possibility in any geographic place in the world. Medical care providers should be trained to recognize and manage emerging health threats that may be associated with climate change. Second, there is a scarcity of literature on the impact of climate change on psychosocial and mental health problems. Researchers need to focus and direct their future research to fill this gap. People living under diseases outbreaks are psychologically and mentally fragile and solutions for such problems need to be investigated and developed. Third, research on public health policies, solutions, laws, legislations, and adaptations are highly needed. Fourth, the development and inventions that can minimize the risk of climate change should be encouraged and funded. Such inventions need to be directed toward new engineering systems that can lower emissions and minimize global warming without negatively affecting the economy. Fifth, climate research and solution in developed countries is not enough to solve the global clime crisis. Climate change is a global problem and developed countries need to help and collaborate with other less developed countries to solve climate change problems since the spread of certain diseases is not limited by country borders and because the less developed countries contributed the least to climate change problem. Sixth, research on monitoring, detection, screening, and early warning systems for infectious diseases should be a national and international priority. Finally, governments need to invest sufficient funds for research on innovative solutions for climate change.

### Limitation

The current study has a few limitations. The literature investigated has been retrieved from journals indexed in Scopus while grey literature and publications in non-indexed journals have not been analyzed. Therefore, journals from non-English speaking countries might be underestimated. This has further consequences on the top ten active countries, institutions and authors. The second limitation was the method for counting the number of documents for each country or author or institution. Scopus makes all analysis based on the number of different affiliations in the documents. Therefore, a document with several authors having the same country affiliation was counted once for that country. However, a document with two authors having two different country affiliations were counted once for each country. This has increased the research output of certain countries with greater international research collaboration even if the authors from that country was not the main or corresponding author. The citation analysis did not take into consideration the self-citations which could create a bias in the number of citations for countries, journals, and authors. Finally, the search query was built to focus on climate change and human health. The definition and scope of human health and climate change are broad and complex. Therefore, it is difficult to ensure a 100% inclusion of literature on both topics. However, the author did his best to include all relevant literature with minimum irrelevant documents. The final point is the inclusion of air pollution and air pollutants in the search query. The author included these keywords with restrictions to keep the manuscript focused on climate change. The purpose of including these terms was to retrieve documents discussing air pollution and health within the context of climate change. Therefore, the number of documents retrieved in this topic was presented by research theme 4 (cluster 4) which was the smallest cluster. Inclusion of air pollution in the search query without restriction will retrieve large volume of irrelevant documents on pollution that were irrelevant to climate change.

## Conclusion

This was the first bibliometric study on climate change and health or infection-related literature. Key players, research themes, and research gaps were identified. The current study provided researchers and policymakers with baseline data in this field. The current study emphasized the importance of climate change on the epidemiology and geography of infectious diseases. Adaptive national and international measures to combat climate change should include plans to contain the expected increase in vector-borne diseases particularly malaria and dengue. The current study showed inadequate international research collaboration which is highly needed for countries in EMRO, AFRO, and SEARO regions. Finally, national and international health organizations should encourage and fund researchers to do continuous assessment and research on the impact of climate change on various health aspects and on various types of infections.

## Supplementary information

**Additional file 1.** Search strategy and keywords for documents on climate change and health (health-related literature).

## Data Availability

All data presented in this manuscript are available on Scopus database using the search query listed in the methodology section.

## Abbreviations

WHO: World Health Organization
CDC: Centers for Disease Control and Prevention
Q1: First Quartile
IPCC: Intergovernmental Panel on Climate Change
UNFCCC: United Nations Framework Convention on Climate Change
