# Supplementary material for: Bibliometric analysis of peer-reviewed literature on climate change and human health with an emphasis on infectious diseases
Source: Global Health. 2020 May 8;16:44. doi: 10.1186/s12992-020-00576-1 (PMC7206222; doi:10.1186/s12992-020-00576-1)
Supplement: Supplementary file 1 — Additional file 1. Search strategy and keywords for documents on climate change and health (health-related literature). [file 12992_2020_576_MOESM1_ESM.docx]

**Bibliometric analysis of peer-reviewed literature on climate change and human health with an emphasis on infectious diseases**

**Additional file 1**: Search strategy and keywords for documents on climate change and health (**health-related literature**)

In the first step, a search query for infectious diseases was carried out

In the second step, documents with titles on climate change and health– related keywords but not related to infections were retrieved

In the third step, documents with titles related to climate change and published in health-related journals were retrieved

The sum of the three steps gave the overall number of documents on climate change and health (**health-related literature**)

| **Step** | **Health-related search query (Overall literature)** |
| --- | --- |
| **1** | **The search query for infection related literature = 1207 (See core literature for keywords)** |
| **2** | TITLE ( *"Climat* Chang*"*  OR  *"greenhouse effect"*  OR  *"changing climate"*  OR  *"global warming"*  OR  *"extreme weather"*  OR  *"climate variability"*  OR  *"greenhouse gas"*  OR  *"rising temperature"*  or "heat wave*")  OR  ( TITLE ( *flood*  OR  *drought*  OR  *precipitation*  OR  *"extreme temp*"*  OR  *heatwave** )  *AND  TITLE-ABS ( "climat* chang*" or "air pollut*") AND TITLE-ABS("climat* change") )  AND  TITLE ( health OR illness OR morbidity OR mortality OR disease OR injury OR death OR infant OR children OR pregna* OR "Non-communicable diseases" OR cancer OR "Cardiovascular disease" OR diabet* OR hypertension OR "Myocardial infarction" OR "Cerebrovascular disease" OR stroke OR respiratory OR "Mental health" OR depression OR schizophrenia OR neurological OR "Stress-Related disorders" OR asthma OR allerg* OR airway OR "Food insecurity" OR "Food security" OR hunger OR malnutrition OR nutrition OR cyanide OR hepatitis OR trauma OR ptsd OR asthma OR respiratory OR allergen OR health OR "cardiac disease*" OR skin OR dermatology* OR infertility OR psychology* or brain or alzheim* or diabet* or hypertens* or mental or neurolog* or mood or psychiatry or psychiatric or psychology or dermatolog* or skin or eye or bone or orthopedic or dependence or abuse or addiction) AND NOT  TITLE-ABS ( avian  OR  bird  OR  carbon  OR  botany  OR  phyto  OR  forest  OR  plant*  OR  amphi*  OR  tree  OR  marine  OR  aquatic  OR  plants  OR  trees  OR  animal  OR  aquatic  OR  bluetongue  OR  sheep  OR  wildlife  OR  soil  OR  aedes  OR  chytridiomyc*  OR  marine  OR  mangrove  OR  shrub  OR  dog )  AND NOT  SRCTITLE ( soil  OR  hydrology  OR  marine  OR  forest )  AND NOT  SRCTITLE ( soil  OR  hydrology  OR  forest* )  AND  ( LIMIT-TO ( SRCTYPE ,  "j" ) )  AND  ( EXCLUDE ( DOCTYPE ,  "er" ) )  AND  ( EXCLUDE ( EXACTSRCTITLE ,  "Journal Of Plant Diseases And Protection" ) )  AND  ( EXCLUDE ( EXACTKEYWORD ,  "Chytrid Fungus" )  OR  EXCLUDE ( EXACTKEYWORD ,  "Chytridiomycetes" )  OR  EXCLUDE ( EXACTKEYWORD ,  "Chytridiomycosis" )  OR  EXCLUDE ( EXACTKEYWORD ,  "Chytridiomycota" ) )* |
| **3** | TITLE-ABS ( "Climat* Change" OR "greenhouse effect" OR "changing climate" OR "global warming" OR "extreme weather" OR "climate variability" OR "greenhouse gas" OR "rising temperature" or "heat wave" ) OR ( TITLE (flood OR drought OR precipitation OR temperature* OR warm* or "air pollut*") AND TITLE-ABS ( "climat* chang*" ) ) AND  SRCTITLE ( *"public health"*  OR  *nurs**  OR  *medicine*  OR  *disease*  OR  *clinical*  OR  *"global health"*  OR  *"food security"*  OR  *respiratory*  OR  *allergy*  OR  *asthma*  OR  *dermatology*  OR  *oncology* ) |
| **4** | **#1 OR #2 OR #3 = 4247** |

**Bibliometric analysis of peer-reviewed literature on climate change and human health with an emphasis on infectious diseases**

**Additional file 1**: Search strategy and keywords for documents on climate change and infectious diseases (**infection-related literature**)

In the first step, all documents with titles on climate change and published in infection – related journals were retrieved

In the second step, we retrieved all documents with titles on climate change and infections

In the third step, we retrieved all documents with title/abstract on climate change that were published in infection-related journals and having titles with infection – related keywords

**The sum of the three steps gave the total number of documents on climate change and infectious diseases (infection-related literature)**

| **Step** | **Infection – related keywords** |
| --- | --- |
| **1** | TITLE ( "Climat* Change"  OR  "greenhouse effect"  OR  "changing climate"  OR  "global warming"  OR  "extreme weather"  OR  "climate variability"  OR  "greenhouse gas"  OR  "rising temperature" )  OR  ( TITLE ( flood  OR  drought  OR  precipitation  OR  temperature*  OR  warm* )  AND  TITLE-ABS ( "climat* chang*" ) )  AND  ( SRCTITLE ( infectious  OR  infection  OR  malaria  OR  "tropical diseases"  OR  "parasite"  OR  virology  OR  "tropical medicine"  OR  virus ) )  AND  ( EXCLUDE ( PUBYEAR ,  2019 ) )  AND  ( LIMIT-TO ( SRCTYPE ,  "j" ) )  AND  ( EXCLUDE ( DOCTYPE ,  "er" ) ) |
| **2** | TITLE ( *"Climat* Change"*  OR  *"greenhouse effect"*  OR  *"changing climate"*  OR  *"global warming"*  OR  *"extreme weather"*  OR  *"climate variability"*  OR  *"greenhouse gas"*  OR  *"rising temperature"* )  OR  ( TITLE ( *flood*  OR  *drought*  OR  *precipitation*  OR  *temperature**  OR  *warm** )  AND  TITLE-ABS ( *"climat* chang*"* ) )  AND  ( TITLE ( *"Climat* Chang*"*  OR  *"greenhouse effect"*  OR  *"changing climate"*  OR  *"global warming"*  OR  *"extreme weather"*  OR  *"climate variability"*  OR  *"greenhouse gas"*  OR  *"rising temperature"* )  OR  ( TITLE ( *flood*  OR  *drought*  OR  *precipitation*  OR  *temperature*  OR  *warm*  OR  *rainf** )  AND  TITLE-ABS ( *"climat* chang*"* ) )  AND  TITLE ( *"waterborne disease*"*  OR  *"west nile virus"*  OR  *ebola*  OR  *babesiosis*  OR  *"red tides"*  OR  *hantavirus*  OR  *hiv*  OR  *"immunodef* virus"*  OR  *"rift valley fever"*  OR  *"h*emorrhagic fever"*  OR  *helminth**  OR  *"mosquito borne"*  OR  *"common cold"*  OR  *amebia**  OR  *myiasis*  OR  *ascariasis*  OR  *pediculosis*  OR  *scabies*  OR  *toxoplasmosis*  OR  *"parasite"*  OR  *candida**  OR  *malaria*  OR  *tuberculosis*  OR  *zika*  OR  *influenza*  OR  *herpes*  OR  *"avian *flu*"*  OR  *"swine *flu*"*  OR  *helminth**  OR  *zoonotic*  OR  *brucellosis*  OR  *pneumonia*  OR  *schistosomiasis*  OR  *buruli*  OR  *lyme*  OR  *toxoplas**  OR  *leptospirosis*  OR  *pediculosis*  OR  *chagas*  OR  *rabies*  OR  *coronavirus*  OR  *zoonosis*  OR  *"resp* tract infect*"*  OR  *cytomegalo**  OR  *"tropical disease*"*  OR  *measles*  OR  *mumps*  OR  *pneumonia*  OR  *echinococcosis*  OR  *meningitis*  OR  *cholera*  OR  *chikungunya*  OR  *rubella*  OR  *smallpox*  OR  *filariasis*  OR  *"Vector-borne"*  OR  *leprosy*  OR  *poliomy**  OR  *taeniasis*  OR  *cysticercosis*  OR  *trypanos**  OR  *varicella*  OR  *acinetobacter*  OR  *strongyloid**  OR  **infection*  OR  *giardia*  OR  *trypano**  OR  *"japanese enceph*"*  OR  *hepatit**  OR  *"SARS"*  OR  *shigella*  OR  *taenia*  OR  *toxoplasm**  OR  *pneumonia*  OR  *anthrax*  OR  *cryptococcosis*  OR  *rubella*  OR  *plague*  OR  *chikungunya*  OR  *mers-cov*  OR  *tularaemia*  OR  *infection*  OR  *infectious*  OR  *dengue*  OR  *"parasite"*  OR  *"communicable diseases"*  OR  *"neglected tropical diseases"*  OR  *"yellow fever"*  OR  *plague*  OR  *arbovir**  OR  *infection*  OR  *diarrh**  OR  *waterborne*  OR  *trachoma*  OR  *malaria*  OR  *schistosomiasis*  OR  *"Typhoid fever"*  OR  *amoebiasis*  OR  *cholera*  OR  *hepatitis*  OR  *salmonell**  OR  *shigellosis*  OR  *dengue*  OR  *onchocerciasis*  OR  *"Japanese encephalitis"*  OR  *scabies*  OR  *conjunctivitis*  OR  *"Scrub typhus"*  OR  *leptospirosis*  OR  *"Communicable diseases"*  OR  *norovirus*  OR  *cryptosporidium*  OR  *"Entamoeba histolytica"*  OR  *giardia*  OR  *shigella*  OR  *salmonella*  OR  *campylobacter*  OR  *"Escherichia coli"*  OR  *"tick-borne"*  OR  *"Borrelia burgdorferi"*  OR  *"encephalitis virus"*  OR  *plasmodium*  OR  *leptospirosis*  OR  *kala-azar*  OR  *virus*  OR  *"water borne"*  OR  *"air borne"*  OR  *"food borne"*  OR  *"vector borne"*  OR  *plasmodium*  OR  *anophole**  OR  *"rodent-borne"*  OR  *hantavirus** )  AND NOT  TITLE-ABS ( *plants*  OR  *trees*  OR  *fish*  OR  *animal*  OR  *aquatic*  OR  *ieee*  OR  *bluetongue*  OR  *sheep*  OR  *wildlife*  OR  *soil*  OR  *chytridiomyc**  OR  *parasitism*  OR  *"carbon monoxide"* ) )  AND NOT  SRCTITLE ( *soil*  OR  *hydrology*  OR  *comparative* )  AND  ( EXCLUDE ( PUBYEAR ,  *2019* ) )  AND  ( EXCLUDE ( DOCTYPE ,  *"er"* ) )  AND  ( LIMIT-TO ( SRCTYPE ,  *"j"* ) )  AND  ( EXCLUDE ( PUBYEAR ,  *1963* )  OR  EXCLUDE ( PUBYEAR ,  *1898* ) ) |
| **3** | TITLE-ABS ( *"Climat* Change"*  OR  *"greenhouse effect"*  OR  *"changing climate"*  OR  *"global warming"*  OR  *"extreme weather"*  OR  *"climate variability"*  OR  *"greenhouse gas"*  OR  *"rising temperature"* )  OR  ( TITLE ( *flood*  OR  *drought*  OR  *precipitation*  OR  *temperature**  OR  *warm** )  AND  TITLE-ABS ( *"climat* chang*"* ) )  AND  SRCTITLE ( *infectious*  OR  *infection*  OR  *malaria*  OR  *"tropical diseases"*  OR  *"parasite"*  OR  *virology*  OR  *"tropical medicine"*  OR  *virus* )  AND  TITLE ( *"waterborne disease*"*  OR  *"west nile virus"*  OR  *ebola*  OR  *babesiosis*  OR  *"red tides"*  OR  *hantavirus*  OR  *hiv*  OR  *"immunodef* virus"*  OR  *"rift valley fever"*  OR  *"h*emorrhagic fever"*  OR  *helminth**  OR  *"mosquito borne"*  OR  *"common cold"*  OR  *amebia**  OR  *myiasis*  OR  *ascariasis*  OR  *pediculosis*  OR  *scabies*  OR  *toxoplasmosis*  OR  *"parasite"*  OR  *candida**  OR  *malaria*  OR  *tuberculosis*  OR  *zika*  OR  *influenza*  OR  *herpes*  OR  *"avian *flu*"*  OR  *"swine *flu*"*  OR  *helminth**  OR  *zoonotic*  OR  *brucellosis*  OR  *pneumonia*  OR  *schistosomiasis*  OR  *buruli*  OR  *lyme*  OR  *toxoplas**  OR  *leptospirosis*  OR  *pediculosis*  OR  *chagas*  OR  *rabies*  OR  *coronavirus*  OR  *zoonosis*  OR  *"resp* tract infect*"*  OR  *cytomegalo**  OR  *"tropical disease*"*  OR  *measles*  OR  *mumps*  OR  *pneumonia*  OR  *echinococcosis*  OR  *meningitis*  OR  *cholera*  OR  *chikungunya*  OR  *rubella*  OR  *smallpox*  OR  *filariasis*  OR  *"Vector-borne"*  OR  *leprosy*  OR  *poliomy**  OR  *taeniasis*  OR  *cysticercosis*  OR  *trypanos**  OR  *varicella*  OR  *acinetobacter*  OR  *strongyloid**  OR  **infection*  OR  *giardia*  OR  *trypano**  OR  *"japanese enceph*"*  OR  *hepatit**  OR  *"SARS"*  OR  *shigella*  OR  *taenia*  OR  *toxoplasm**  OR  *pneumonia*  OR  *anthrax*  OR  *cryptococcosis*  OR  *rubella*  OR  *plague*  OR  *chikungunya*  OR  *mers-cov*  OR  *tularaemia*  OR  *infection*  OR  *infectious*  OR  *dengue*  OR  *"parasite"*  OR  *"communicable diseases"*  OR  *"neglected tropical diseases"*  OR  *"yellow fever"*  OR  *plague*  OR  *arbovir**  OR  *infection*  OR  *diarrh**  OR  *waterborne*  OR  *trachoma*  OR  *malaria*  OR  *schistosomiasis*  OR  *"Typhoid fever"*  OR  *amoebiasis*  OR  *cholera*  OR  *hepatitis*  OR  *salmonell**  OR  *shigellosis*  OR  *dengue*  OR  *onchocerciasis*  OR  *"Japanese encephalitis"*  OR  *scabies*  OR  *conjunctivitis*  OR  *"Scrub typhus"*  OR  *leptospirosis*  OR  *"Communicable diseases"*  OR  *norovirus*  OR  *cryptosporidium*  OR  *"Entamoeba histolytica"*  OR  *giardia*  OR  *shigella*  OR  *salmonella*  OR  *campylobacter*  OR  *"Escherichia coli"*  OR  *"tick-borne"*  OR  *"Borrelia burgdorferi"*  OR  *"encephalitis virus"*  OR  *plasmodium*  OR  *leptospirosis*  OR  *kala-azar*  OR  *virus*  OR  *"water borne"*  OR  *"air borne"*  OR  *"food borne"*  OR  *"vector borne"*  OR  *plasmodium*  OR  *anophole**  OR  *"rodent-borne"*  OR  *hantavirus** )  AND  ( EXCLUDE ( PUBYEAR ,  *2019* )  OR  EXCLUDE ( PUBYEAR ,  *1975* )  OR  EXCLUDE ( PUBYEAR ,  *1964* ) )  AND  ( LIMIT-TO ( SRCTYPE ,  *"j"* ) )  AND  ( EXCLUDE ( DOCTYPE ,  *"er"* ) ) |
| **4** | **#1 OR #2 OR #3 = 1207** |
